# Effects of diet and exercise interventions on diabetes risk factors in adults without diabetes: meta-analyses of controlled trials

**DOI:** 10.1186/1758-5996-6-127

**Published:** 2014-11-24

**Authors:** J A D Ranga Niroshan Appuhamy, Ermias Kebreab, Mitchell Simon, Rickey Yada, Larry P Milligan, James France

**Affiliations:** Department of Animal and Poultry Science, Centre for Nutrition Modelling, University of Guelph, Guelph, N1G 2 W1 Ontario Canada; Department of Animal Science, University of California, One Shield Avenue, Davis, CA 95616 USA; Faculty of Land and Food Systems, University of British Columbia, Vancouver, V6T 1Z4 Canada

## Abstract

**Background and aims:**

Fasting insulin (FI), fasting glucose (FG), systolic blood pressure (SBP), high density lipoproteins (HDL), triacylglycerides (TAG), and body mass index (BMI) are well-known risk factors for type 2 diabetes. Reliable estimates of lifestyle intervention effects on these factors allow diabetes risk to be predicted accurately. The present meta-analyses were conducted to quantitatively summarize effects of diet and exercise intervention programs on FI, FG, SBP, HDL, TAG and BMI in adults without diabetes.

**Materials and methods:**

MEDLINE and EMBASE were searched to find studies involving diet plus exercise interventions. Studies were required to use adults not diagnosed with type 2 diabetes, involve both dietary and exercise counseling, and include changes in diabetes risk factors as outcome measures. Data from 18, 24, 23, 30, 29 and 29 studies were used for the analyses of FI, FG, SBP, HDL, TAG and BMI, respectively. About 60% of the studies included exclusively overweight or obese adults. Mean age and BMI of participants at baseline were 48 years and 30.1 kg/m^2^. Heterogeneity of intervention effects was first estimated using random-effect models and explained further with mixed-effects models.

**Results:**

Adults receiving diet and exercise education for approximately one year experienced significant (*P* <0.001) reductions in FI (-2.56 ± 0.58 mU/L), FG (-0.18 ± 0.04 mmol/L), SBP (-2.77 ± 0.56 mm Hg), TAG (-0.258 ± 0.037 mmol/L) and BMI (-1.61 ± 0.13 kg/m^2^). These risk factor changes were related to a mean calorie intake reduction of 273 kcal/d, a mean total fat intake reduction of 6.3%, and 40 minutes of moderate intensity aerobic exercise four times a week. Lifestyle intervention did not have an impact on HDL. More than 99% of total variability in the intervention effects was due to heterogeneity. Variability in calorie and fat intake restrictions, exercise type and duration, length of the intervention period, and the presence or absence of glucose, insulin, or lipid abnormalities explained 23-63% of the heterogeneity.

**Conclusions:**

Calorie and total fat intake restrictions coupled with moderate intensity aerobic exercises significantly improved diabetes risk factors in healthy normoglycemic adults although normoglycemic adults with glucose, insulin, and lipid abnormalities appear to benefit more.

## Introduction

High prevalence of type 2 diabetes is strongly associated with obesity and lack of physical activity. Type 2 diabetes is a major cause of kidney failure, lower-limb amputation, blindness, heart disease and is a leading cause of death among adults in Western countries [1]. Consequently, diabetes creates a major financial burden on national healthcare systems representing, for example, more than 10% of total healthcare expenditures in the USA, Canada and Europe [2].

Type 2 diabetes is a predictable and preventable disease [3]. In addition to pharmacological interventions, type 2 diabetes can be effectively prevented or delayed by lifestyle changes targeting diet and physical activity improvements [4]. Increases in fasting insulin (**FI**), fasting glucose (**FG**), systolic blood pressure (**SBP**), high density lipoproteins (**HDL**), triacylglycerides (**TAG**), and body mass index (**BMI**) are associated with increased risk of developing diabetes and are often used in mathematical models for predicting the risk of developing diabetes [5–7]. Reliable estimates of risk factor responses to lifestyle interventions can improve the accuracy of diabetes risk predictions and assist in planning effective diabetes prevention programs.

Several published studies have investigated the effects of diet and exercise interventions on diabetes risk factors but the estimated effect sizes are inconsistent across studies. Meta-analyses are widely used to compare and combine treatment effects across studies and achieve consensus about the overall treatment effect size. However, conclusions drawn from combining data can be misleading, especially if the individual studies and datasets are considerably different. Therefore, estimation and explanation of the between-study variability or heterogeneity of effect sizes should be an important goal in undertaking meta-analyses. Meta-analyses using random-effect models assume that the studies are a random sample of the entire population of studies, allowing inferences to be generalized beyond the studies included. Random-effect meta-analyses also allow for estimation and exploration of heterogeneity [8].

Meta-analyses have previously been published on lifestyle intervention effects in adults diagnosed with type 2 diabetes [9, 10]. However, the present study focused on effects among individuals without diabetes as these are directly related to diabetes prevention rather than management. Yamaoka and Tango [11], Gillies et al. [12] and Norris et al. [13] published meta-analyses summarizing the efficacy of diet and exercise interventions among adults with pre-diabetes or impaired glucose tolerance (**IGT**). We decided to focus on lifestyle intervention effects irrespective of pre-diabetic risk categories. Evidence suggesting the need for lower cutoff levels for diabetes risk categories in Western populations [14] and including more studies in the analyses provided the impetus for this decision. Besides obtaining estimates of effect sizes for overall lifestyle intervention, we were also interested in examining the effects of important intervention aspects such as calorie and fat intake restrictions and improvements in frequency and duration of exercise.

The objectives of the present meta-analyses were to quantitatively summarize 1) overall effect size of lifestyle education programs targeting both diet and physical exercise modifications and 2) effects of important dietary and exercise attributes on FI, FG, SBP, HDL, TAG and BMI in adults without diabetes in Western populations.

## Methods

### Literature search

**Figure 1:**
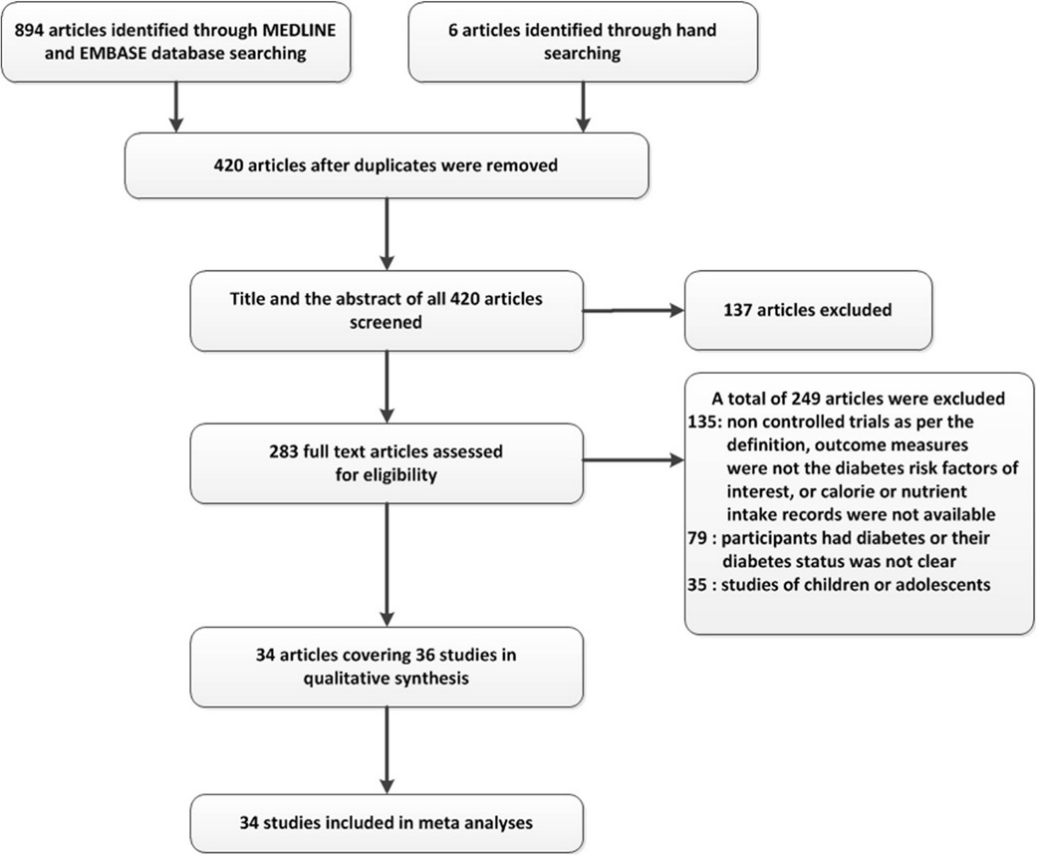
**A schematic representation summarizing the literature search.**

Studies involving both diet and physical exercise interventions where FI, FG, SBP, TAG, HDL and/or BMI were major outcome measures were searched. Study participants were required to be from Western populations, where the majority is Caucasian. This distinction was made to minimize the potential heterogeneity of intervention effects due to ethnic differences. Study participants in the control group were required to continue with their regular exercise and dietary habits and not receive any diet or exercise counseling prior to or during the study period. MEDLINE and EMBASE computer searches [15] were carried out for articles describing human clinical trials published in English before June 30, 2012 using the keywords: “diet or weight loss”, “exercise or physical activity”, “diabetes risk or cardiovascular risk”, “obese or overweight” and “men and women”. Different combinations of key words were searched in both MEDLINE and EMBASE (Table 1). A total of 894 (278 with MEDLINE and 616 with EMBASE) studies were retrieved from the computer searches. Six hand-searched articles were additionally included (Figure 1). 474 duplicates were removed leaving 420 records to be screened. Two authors separately screened the abstracts of the 420 articles and excluded 137 articles because they were about surveys, feasibility studies, trial designs or mathematical and statistical model analyses (Figure 1). Moreover, some of the excluded studies were not from Western countries. The remaining 283 full text articles were assessed for eligibility to be included. The eligibility criteria were 1) trials included adult (men and/or women) participants who were not diagnosed with diabetes, 2) intervention involved both dietary and exercise counseling, 3) dietary counseling targeted calorie and macronutrient intake modifications, 4) outcome measures included changes in diabetes risk factors compared to a control group, 5) availability of mean and variance measures of risk factor changes from baseline in both control and intervention groups, and 6) availability of information on macronutrient intake changes of intervention and control participants. A total of 249 articles were excluded as they did not meet the eligibility criteria (Figure 1). Two [16, 17] of the 34 remaining articles reported risk factors for men and women separately. The male and female groups in these reports were considered two separate studies leaving 36 studies for quality assessment. Quality of the selected studies was evaluated by assessing the risk of four biases [18]: 1) selection bias representing systematic differences between baseline characteristics of intervention and control groups, 2) performance bias regarding exposure to factors other than the interventions of interest, 3) attrition bias involving systematic differences between groups in withdrawals from a study, and 4) publication bias assessed as described below. Baseline characteristics of study participants were not considerably different between control and intervention arms in any of the 36 studies. Although some studies encouraged increased fiber intake and reduced cholesterol intake besides macronutrient intake modifications, these studies were kept in the dataset because fiber and cholesterol intake effects can be accounted for in the analyses as described below. Most of the studies reported no systematic differences between withdrawal groups. However, two articles were excluded because the studies had considerable dropout rates (>30%) with no note on the similarities or differences of the withdrawal groups (Figure 1). Although, the statistical significance (*P*-values) of the intervention effects did not change, the magnitude of the effect sizes changed notably when these two studies were excluded. Since obtaining accurate effect size estimates for lifestyle interventions regarding diabetes risk was the main objective of the present study, we proceeded with the meta-analyses without these two studies. Therefore, the final data set used for the meta-analyses contained 34 studies (Figure 1 and Table 2).

**Table 1:** **Number of articles retrieved from MEDLINE and EMBASE databases for different key word combinations**

Keywords	MEDLINE	EMBASE
Diet, exercise, obese, diabetes risk, men, women	27	56
Diet, exercise, overweight, diabetes risk, men, women	29	50
Diet, exercise, obese, cardiovascular risk, men, women	32	63
Diet, exercise, overweight, cardiovascular risk, men, women	34	69
Diet, physical activity, obese, diabetes risk, men, women	29	67
Diet, physical activity, overweight, diabetes risk, men, women	28	84
Diet, physical activity, obese, cardiovascular risk, men, women	33	77
Diet, physical activity, overweight, cardiovascular risk, men, women	31	97
Weight loss, exercise, obese, diabetes risk, men, women	18	21
Weight loss , exercise, overweight, diabetes risk, men, women	17	32

### Data and variables

Data were extracted and put onto structured Excel datasheets designed to capture relevant information in a concise manner. Two authors conducted the screening and data extraction independently using a common set of instructions prepared for the relevant tasks. Sample size (N), mean risk factor levels at baseline and standard deviation (SD) of FI, FG, SBP, HDL, TAG and BMI changes during intervention of the control and intervention arms were essential to the analyses. Mean FI and BMI values were recorded in mU/L and kg/m^2^, respectively, while the other risk factors were recorded in mmol/L. In the absence of a reported SD, if 95% confidence intervals for the risk factor changes were provided, the SD was calculated assuming the changes were normally distributed. In a few cases, the SD was calculated using the *P*-values for risk factor changes from baseline and corresponding test statistics. N was the number of participants completing each treatment and undergoing post-treatment risk factor measurements. Information on energy (kcal/d) and the macronutrients: carbohydrate, protein, total fat, and saturated fat intake [% of energy intake (%E)] at baseline and at the end of the interventions were recorded in separate columns of the datasheet. When the macronutrient intakes were reported in g/d (along with daily energy intake), the %E values were calculated using Atwater energy equivalents [19]. Energy and macronutrient intake changes (from baseline) in the control and intervention groups were calculated separately. Difference (*d*) between these changes mean values are given in Table 3 was then calculated and used in the statistical models.

**Table 2:** **Summary description of the diet plus exercise intervention studies included in the analyses**

Study	Authors and year	Country	Age ^1^	Women ^2^	IGT/IR/MetS ^3^	BMI ^4^	Diet intervention guidelines ^5^	Exercise intervention guidelines ^5^
1	Straznicky et al. 2012 [20]	Australia	55	0.41	MS	32.7	~650 kcal/d restriction, 22% protein, 30% TF, 9% SF	aerobic (biking), 40 min/S, 3–4 S/wk
2	Blumenthal et al. 2010 [21]	USA	52	0.69	NA	33.3	~500 kcal/d restriction, 27% TF	aerobic (biking and walking), 40 min/S, 3–4 S/wk
3	Ibanez et al. 2010 [22]	Spain	50	1.00	NA	35.0	500 kcal/d restriction	resistance training, 45–60 min/S, 2 S/wk
4	Straznicky et al. 2010 [23]	Australia	55	0.41	MS	32.4	~500 kcal/d restriction, 30% TF, 9% SF	aerobic (biking), 40 min/S, 3 S/wk
5	Roumen et al. 2008 [24]	Netherlands	56	0.45	IGT	29.4	reduced caloric and reduced fat diet	aerobic plus resistant training, 30 min/S, 5 S/wk
6	Herder et al. 2009 [25]	Finland	56	0.50	IGT	31.2	<30% TF, <10% SF	endurance training, >30 min/S, ~6 S/wk
7	Mosca et al. 2008 [26]	USA	48	0.66	NA	28.1	low-SF and low-cholesterol diet	moderate physical activity (brisk walking), 30–60 min/S
8	Morgan et al. 2009 [27]	UK	41	0.74	NA	31.6	Rosemary Conley's controlled-calorie low-fat diet	Rosemary Conley's Fitness plan with weekly classes
9	Dale et al. 2009 [28]	New Zealand	46	0.67	IR	34.6	400 kcal/d restriction, 27% TF, 9% SF	high intensity training, 30 min/S, 5 S/wk
10	Meckling and Sherfey, 2007 [29]	Canada	43	1.00	NA	29.9	500 kcal/d restriction, high protein (37%) diet	endurance training, 36 min/S, 3 S/wk
11	Burke et al. 2007 [30]	Australia	56	0.56	NA	30.1	<30% TF, <10% SF	moderate intensity, 30 min/S, most days/wk
12	Bo et al. 2007 [31]	Italy	56	0.58	MS	30.0	reduced TF and SF intake	moderate intensity (i. e. brisk walking), ~150 min/wk
13	Arciero et al. 2006 [32]	USA	43	0.48	NA	27.8	high protein (40%) and low fat (20%) diet	resistance and cardiovascular training, 20 min/S, 4–6 S/wk
14	Brekke et al. 2005 [33]	Sweden	42	0.37	NA	26.1	<30% TF intake, <10% SF intake	walking or more intensive exercise, 30 min/S, 4–5 S/wk
15	Watkins et al. 2003 [34]	USA	50	0.50	NA	33.7	500 kcal/d restriction, <20% TF	cycle ergometry and jogging, or walking, ~60 min/S, 3–4 S/wk
16	Lindstrom et al. 2003 [35]	Finland	55	0.66	IGT	31.3	200 kcal/d restriction, <30% TF, <10% SF	endurance exercise & resistance training, >30 min/S
17	Esposito et al. 2003 [36]	Italy	35	1.00	NA	34.5	1400 kcal/d, 55% carbohydrate, 30% TF, <10% SF	aerobic exercise (walking and swimming)
18	Mensink et al. 2003 [37]	Netherlands	56	0.43	IGT	29.5	>55% carbohydrate, <30% TF, <10% SF	moderate physical activity, >30 min/S, 5 S/wk
19	McAuley et al. 2002 [38]	New Zealand	46	0.71	IR	34.5	400 kcal/d restriction, 27% TF, 9% SF	Moderate exercise plus resistance training, >20 min/S, 5 S/wk
20	Miller et al. 2002 [39]	USA	54	0.62	NA	33.7	500 kcal/d restriction, 27% TF, 6% SF	aerobic (brisk walking and biking), 30–45 min/S, 3 S/wk
21	Reseland et al. 2001 [40]	Norway	45	0.00	MS	27.5	400 kcal/d restriction, <30% TF	endurance exercise, 1 h/S, 3 S/wk
22	Oldroyd et al. 2001 [41]	UK	58	0.40	IGT	30.2	<30% TF intake, ~50% carbohydrate	aerobic exercise, 20–30 min/S, 2–3 S/wk
23	Kuller et al. 2001 [42]	USA	47	1.00	NA	25.0	Calorie restriction upto 1300 kcal, 25% TF, 7% SF	increasing physical activity to 1250 kcal expended weekly
24	Ornish et al. 1998 [43]	USA	60	0.09	NA	26.9	10%-fat vegetarian diet	moderate-intensity aerobic, 1 h/S, 5 S/wk
25	Stefanick et al. 1998 (female) [16]	USA	57	1.00	NA	25.6	<30% TF intake, <7% SF intake	aerobic (jogging and brisk walking), 60 min/S, 3 S/wk
26	Stefanick et al. 1998 (male) [16]	USA	48	1.00	NA	27.8	<30% TF intake, <7% SF intake	aerobic (jogging and brisk walking), 60 min/S, 3 S/wk
27	Wing et al. 1998 [44]	USA	46	0.78	NA	36.0	600-700 kcal/d restriction, 20% TF intake	aerobic (brisk walking), 60 min/S, 5 S/wk
28	Simkin-Silverman et al. 1995 [45]	UK	47	1.00	NA	25.1	<25% TF intake, <7% SF intake	brisk walking spending 1000 kcal/wk, 3–5 S/wk
29	Hellenius et al. 1993 [46]	Sweden	46	0.00	NA	25.6	12% daily calorie and 10% TF restriction	aerobic exercise, 30–45 min/S, 2–3 S/wk
30	Svendsen et al. 1993 [47]	Denmark	54	1.00	NA	29.7	800 kcal/d restriction, low fat (25%) diet	aerobic plus resistant training, 75 min/S, 3 S/wk
31	Page et al. 1993 [48]	UK	40	0.23	IGT	25.9	50-55% carbohydrate, 30% TF, high fiber	aerobic weight and circuit training, swimming, >3 S/wk
32	Schuler et al. 1992 [49]	Germany	54	0.00	NA	26.6	<20% TF, 65% carbohydrate, PUSF: SF ratio >1.0	daily exercise (75% MHR), >30 min/S
33	Wood et al. 1991 (male) [17]	USA	38	0.00	NA	33.5	55% carbohydrate, 30% TF, <10% SF	aerobic (brisk walking and jogging), 35 min/S, 3 S/wk
34	Wood et al. 1991 (female) [17]	USA	38	1.00	NA	26.3	55% carbohydrate, 30% TF, <10% SF	aerobic (brisk walking and jogging), 35 min/S, 3 S/wk

^1^Average age of participants at baseline (years), ^2^fraction of women participants, ^3^whether participants had abnormalities such as impaired glucose tolerance (IGT), insulin resistance (IR) and metabolic syndrome (MetS) or no abnormalities (NA), ^4^average BMI of participants at baseline (kg/m^2^), ^5^ *TF* = Total fat (% of energy), *SF* = Saturated fat (% of energy), and *S* = exercise session.



When the nutrient intake changes of control participants were not available, they were assumed to be unchanged from baseline during the interventions. With respect to exercise interventions, two explanatory variables: the number of exercise sessions per week (**ES**) and minutes of exercise per session (**EM**) were created. Two binary variables were also created to represent presence or absence of dietary counseling regarding fiber intake increase (**FB**) and cholesterol intake reduction (**CH**). As some exercise intervention programs included resistance training sessions, a binary variable (**ERT**; 1 = presence or 0 = absence) was created to examine the impact of resistance training on the diabetes risk factors. When actual ES and EM measures were not available, they were assumed to be equivalent to the exercise intervention guidelines (Table 2). Some studies exclusively recruited people with abnormalities such as impaired glucose tolerance (**IGT**), insulin resistance (**IR**), or metabolic syndrome (**MetS**). Another binary variable (**ABN**) was created to identify studies that exclusively used participants with such abnormalities. Average age in years, study duration in months and the fraction of women in the study population (ranging from 0, only men to 1, only women) were also extracted. When the biomarker levels were measured at multiple time points, the measures of the nutrient intake at the latest time point were used. For some studies, the selected time point was after a follow-up period. Therefore, an additional binary variable (**IFU**) was generated to represent presence or absence of a follow-up period.

### Statistical analyses

Separate meta-analyses were conducted for each biomarker using the *metafor* (version 1.6-0) package in R (version 2.12.2) [50]. Mean difference (**MD**) was chosen as the effect size measure as it allows effect size interpretation in the original units of risk factor measurement.


**Figure 2:**
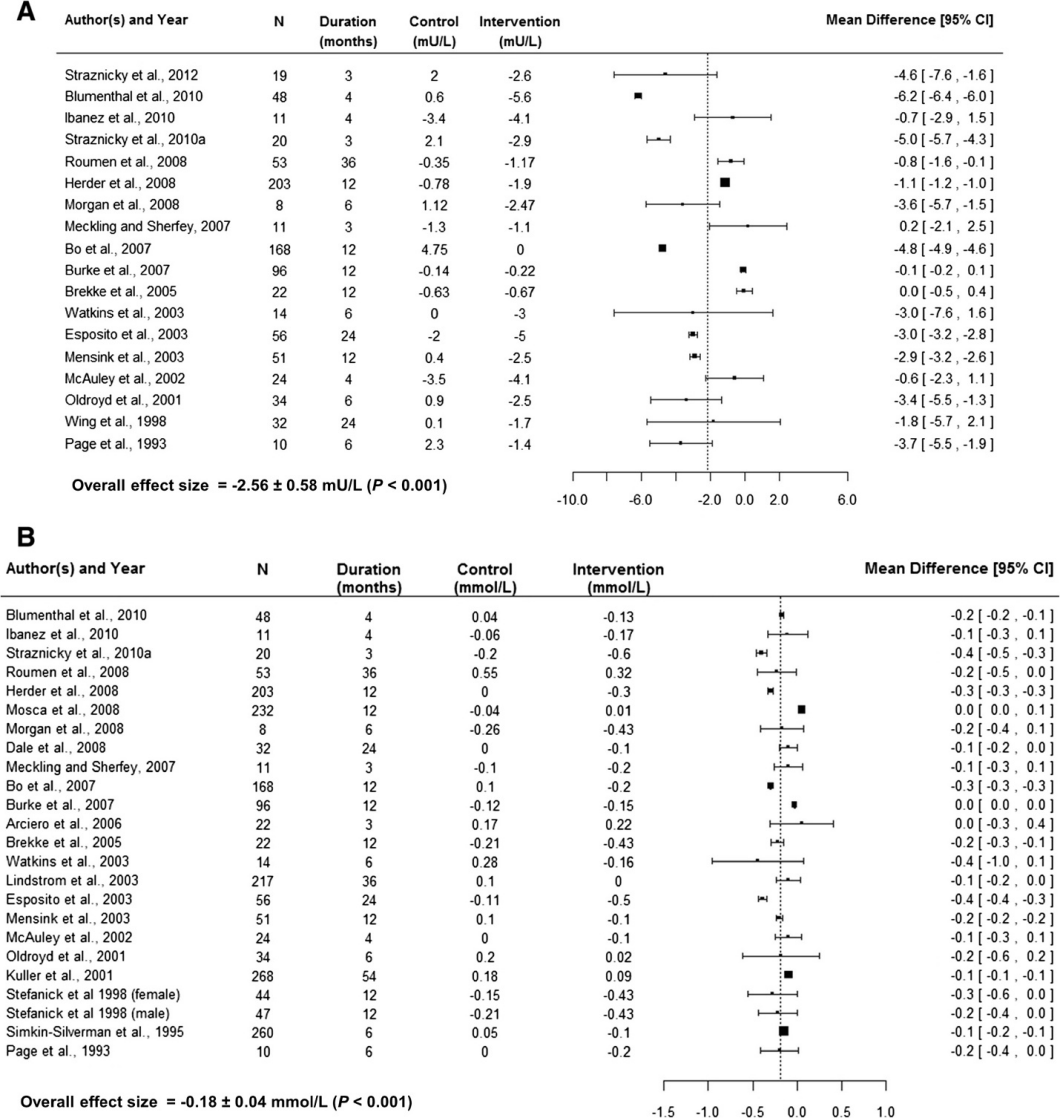
**Forest plots showing absolute fasting insulin (A) and fasting glucose (B) changes (from baseline) in control and intervention arms, average sample size (N) across both arms, time duration related to the changes and mean difference of changes with its confidence interval (95% CI).**

**Figure 3:**
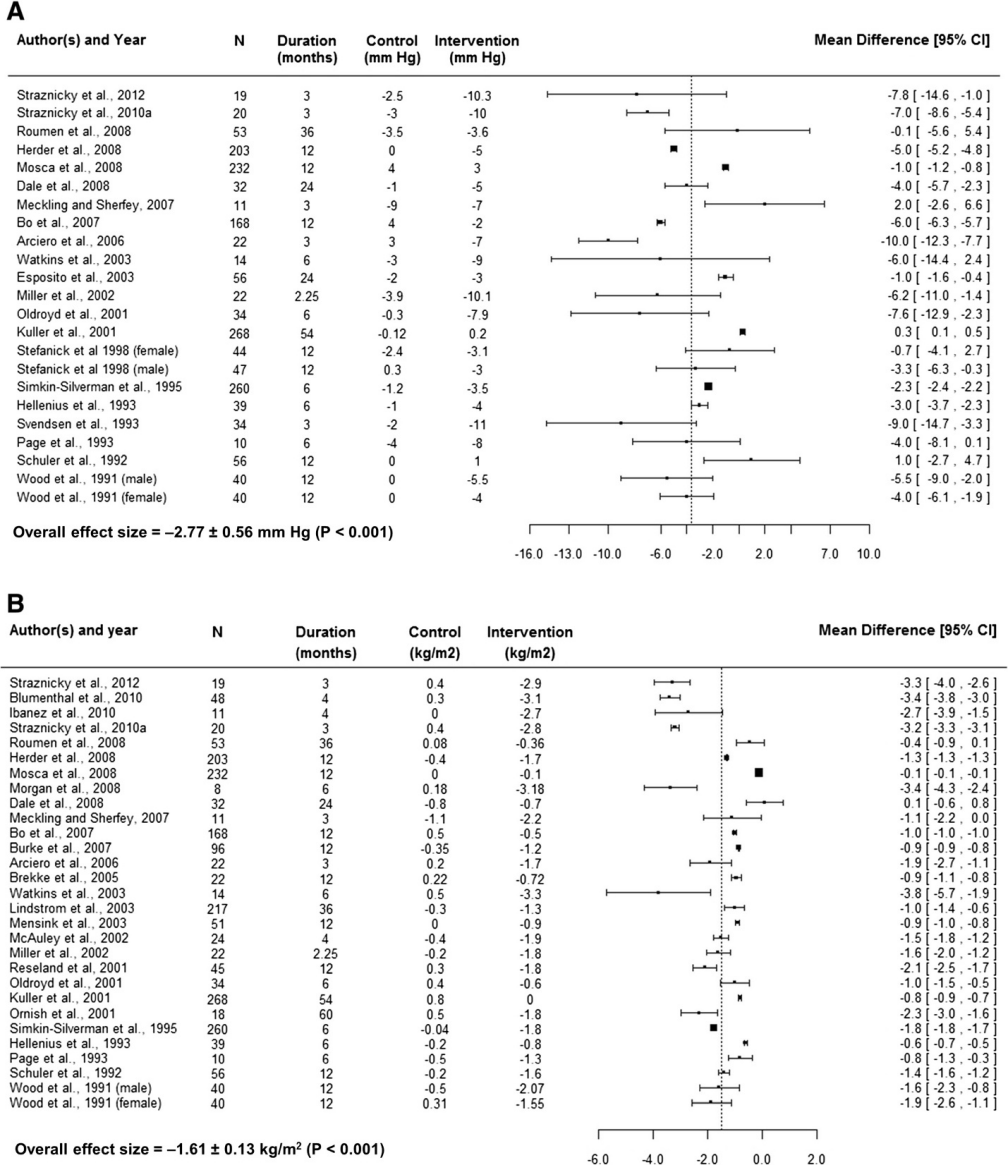
**Forest plots showing absolute systolic blood pressure (A) and body mass index (B) changes (from baseline) in control and intervention arms, average sample size (N) across both arms, time duration related to the changes and mean difference of changes with its confidence interval (95% CI).**

**Figure 4:**
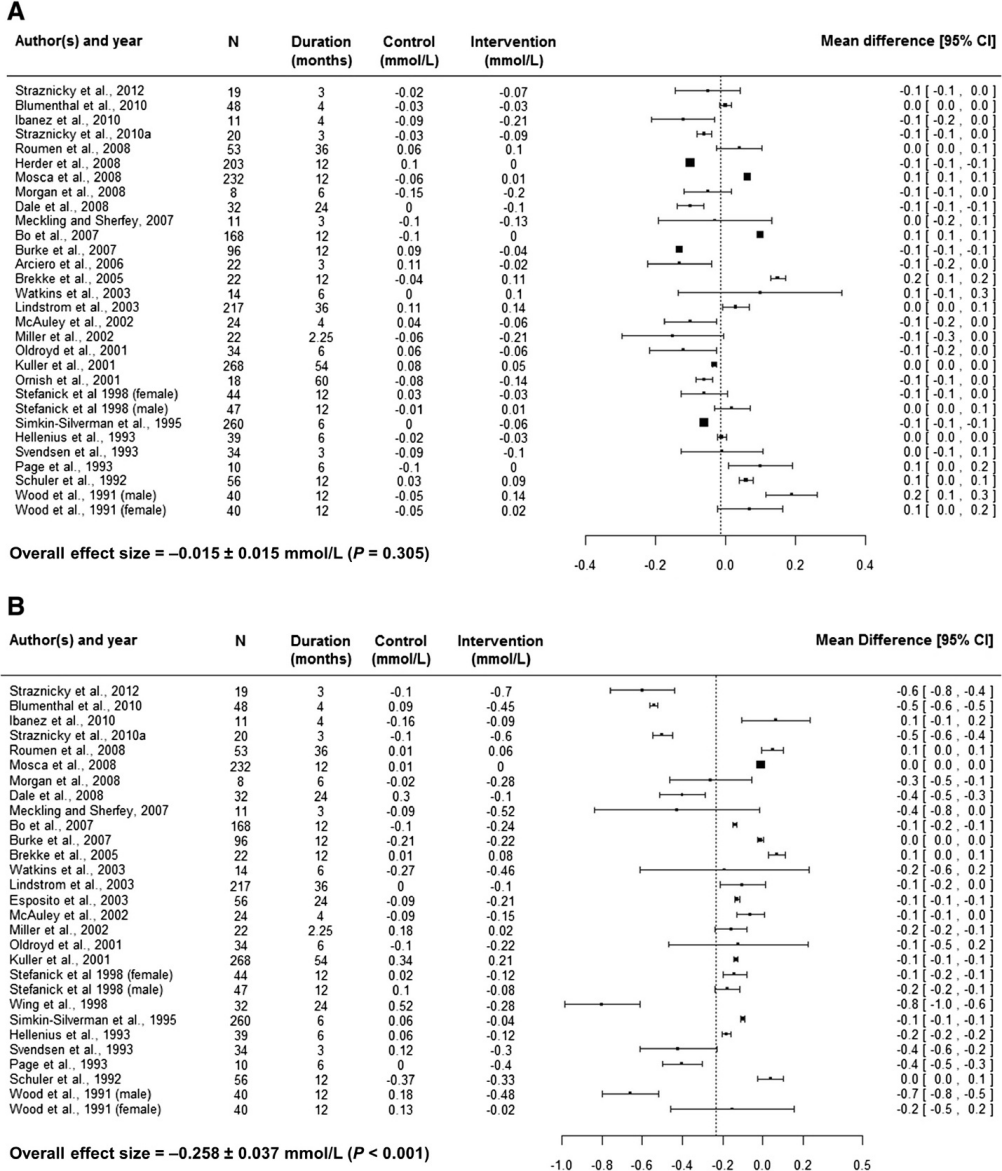
**Forest plots showing absolute high density lipoprotein (A) and triacylglyceride (B) changes (from baseline) in control and intervention arms, average sample size (N) across both arms, time duration related to the changes and mean difference of changes with its confidence interval (95% CI).**

This choice was further supported by the fact that the *metafor* package allows for weighting individual studies for corresponding sample variance as described below [50]. The forest plots of each risk factor were also constructed using MD (Figures 2, 3, and 4). In addition to MD, forest plots give average sample size, intervention duration, and absolute risk factor changes in the control and intervention arms.

Let:


where *y*_*i*_ is the observed effect size or MD in the *i*^th^ study, *θ*_*i*_ is the corresponding (unknown) true effect size, *e*_*i*_ is the sampling error [*e*_*i*_ ~ *N*(0, *v*_*i*_)]. Sampling variances (i.e., *v*_*i*_) are assumed to be known and remained fixed during estimation in order to weight the individual studies when estimating model parameters [50]. Between-study variability (heterogeneity) of the true effects *θ*_*i*_ was assumed to be purely random, leading to random-effect models given by:


where *θ*_*i*_ is the true effect size (e.g., MD) in the *i*^th^ study, *μ* is the overall true effect size, and *u*_*i*_ is the random deviation from the overall effect size (*u*_*i*_ ~ *N*(0, *τ*^2^)), which was unknown but estimated from the data. The true effects were normally distributed with mean *μ* and variance *τ*^*2*^. If *τ*^2^ = 0, homogeneity is implied among true effects across individual studies such that *μ*=*θ*. Heterogeneity (*τ*^2^) was expressed as a percentage of total variability (*τ*^2^ plus sample variance) yielding *I*^*2*^ statistics.

**Table 3:** **Summary statistics for variables across the 34 studies included in the meta-analyses**

Variable	Mean ± SD ^1^	Minimum	Maximum
*Energy and macronutrient intake at baseline*			
Calorie intake, *kcal/d*	2100 ± 260	1583	2900
Carbohydrate, *%E*	46.6 ± 5.0	36.5	58.5
Protein, *%E*	17.2 ± 2.6	12.3	25
Fat, *%E*	34.7 ± 3.3	27.5	43.5
Saturated fat, *%E*	12.9 ± 1.8	9	16.6
*Energy and macronutrient intake changes of intervention participants* ^2^			
Calorie intake, *kcal/d*	-273 ± 169	-828	49.5
Carbohydrate, *%E*	2.9 ± 5.7	-14	11
Protein, *%E*	3.3 ± 5.6	-3.8	21.3
Fat, *%E*	-6.3 ± 3.8	-16.5	0.4
Saturated fat, *%E*	-2.9 ± 2.0	-8	0.0
*Exercise intervention*			
Number of sessions per week	4 ± 1.0	2	7
Session length (minutes)	41 ± 13	20	75
*Risk factor level measurements at baseline*			
FI	14.9 ± 5.4	3.0	24.1
FG	5.55 ± 0.4	4.83	6.20
SBP	128 ± 10	110	143
HDL	1.25 ± 0.2	0.89	1.74
TAG	1.76 ± 0.9	0.85	5.84
BMI	30.2 ± 3.3	25.0	36.0
Age at baseline, years	49 ± 7	35	60
Fraction of women participants	0.59 ± 0.3	0	1
Duration of intervention (months)^3^	14 ± 3.5	2	60

^1^ *SD*, Standard deviation.

^2^Change from baseline compared to that of control participants.

^3^Duration from baseline to the risk factor level measurements used in the analyses.

An *I*^2^ value greater than 50% indicates considerable heterogeneity. Thus, for response variables (e.g., intervention effects on diabetes risk factors in the present study) with *I*^2^ > 50%, the random-effect models were extended to mixed-effect models including fixed effects of explanatory variables (meta-regression analyses) with the potential to explain the heterogeneity in intervention effects. The mixed-effect models were given by:


where *β*_0_ is the overall true effect size, *x*_*ij*_ is the the value of the *j*^th^ explanatory variable (*j* = 1, 2, …, *p*) for the *i*^th^ study, *β*_*j*_ is the change in the true effect size for a unit increase in the *j*^th^ explanatory variable and *u*_*i*_ ~ *N*(0, *τ*^2^). Here, *τ*^2^ denotes the amount of residual heterogeneity [50]. The parameters in the mixed-effect models (*β*_0_, …, *β*_*p*_) were estimated via weighted least squares with weights equal to 1/(*v*_*i*_ + *τ*^2^).

The candidate explanatory variables for the mixed-effect models were energy and macronutrient intake changes, the exercise attributes: ES, EM and ERT, baseline risk factor level, baseline age and BMI, time duration from baseline to the post-intervention risk factor measurements, FB, CH, ABN, IFU and fraction of women in the study population. Each continuous explanatory variable was centered on its mean before being used in the mixed-effect models. This allows for interpreting the meta-regression coefficients in terms of changes in intervention effect size for a unit change in a continuous explanatory variable from its mean. Mixed-effect models including individual explanatory variables were first fitted to the data. Full multivariate mixed-effect models were then formed including all explanatory variables having notable effects (*P* <0.10) when fitted individually. Reduced models were formed via stepwise elimination of one explanatory variable at a time. The final mixed-effect models were chosen by comparing reduced models vs. full models using log-likelihood ratio tests with the maximum likelihood method.

### Publication bias and influence diagnosis

Publication bias and presence of influential cases can affect the validity and robustness of the conclusions from a meta-analysis [51, 52]. Studies for which the effect size estimates and their variability are extremely sensitive were recognized using a leave-one-out approach. The *leave1out* function in metafor package was used to execute this influence diagnostic analysis, which is equivalent to a sensitivity analysis. Cook’s distance and *τ*^*2*^ estimates (*τ*^2^_D_) were obtained when each study was excluded from the data set. [50]. If a study was related to a Cook’s distance >2 and *τ*^2^_D_ two SD below the mean *τ*^2^_D_ of the other studies, that study was removed from the dataset. Initially, 19, 25, 25, 31, 30, and 30 of the 34 studies were chosen for FI, FG, SBP, HDL, TAG and BMI analyses respectively, but after the influence analyses 18, 24, 23, 30, 29, and 29 studies were retained, respectively. Presence of publication bias was assessed using funnel plots. Asymmetric funnel plots indicate the presence of publication bias. Egger’s regression test was used to examine funnel plot asymmetry [50]. None of the funnel plots was found to be significantly asymmetric (*P*-values for funnel plot asymmetry >0.05) indicating absence of publication bias in all cases.

## Results

### Study characteristics

Of the 34 studies included in meta-analyses, 16, 13, and 5 studies were from Europe, North America, and Australia and New Zealand, respectively (Table 2). Information about ethnic composition of participants was not available in all the articles. Based on the articles reporting the ethnic composition, >60% of the study participants were Caucasian. Average age at baseline ranged from 35 to 60 years with a mean of 49 years, indicating that the majority of study participants were middle-aged. Twenty of the 34 studies recruited exclusively overweight and obese individuals. Average baseline BMI varied from 25.0 to 36.0 kg/m^2^ with a mean of 30.2 kg/m^2^ (Table 3). Twelve of the 34 studies examined diet plus exercise intervention specifically in adults with abnormalities such as IGT, IR, or MetS. Average FG of participants in the individual studies ranged between 4.83 and 6.20 mmol/L, with a mean of 5.55 mmol/L. The respective FI range was 3.0 and 24.1 mU/L with a mean of 14.9 mU/L. Time duration from baseline risk factor measurements to the post-intervention measurements varied from 2 to 60 months with a mean of 14 months (Table 3). In a few studies, this duration included a follow-up period in addition to the intervention counselling period. Nine of the studies included only female participants and four used only males while the rest (21 studies) included both males and females. The majority of lifestyle intervention programs aimed to restrict energy intake by 500 kcal/d, total fat ≤30%E, and saturated fat ≤10%E (Table 2) and to improve physical activity through moderate-intensity aerobic exercises such as brisk walking, jogging, cycling, and swimming. About one-third of the intervention programs included instructions for resistance training. Overall, the intervention programs involved an average of 4 exercise sessions a week each lasting 41 minutes (Table 3).

### Heterogeneity

Random-effect model analyses (Table 4) revealed substantial heterogeneity (*τ*^2^, *P* <0.001) of lifestyle intervention effects on all risk factors. The *I*^2^ estimates (*τ*^2^/*τ*^2^ + sample variance) were >99.0% in all cases (Table 4) indicating that between-study variability of intervention effects (*τ*^2^) were >110 times greater than within-study variability (sample variance). Therefore the random-effect models were extended to mixed-effect models to explore heterogeneity.

**Table 4:** **Heterogeneity (** ***τ*** ^***2***^ **) of lifestyle intervention effects and statistical significance of funnel plot asymmetry from random-effect models**

Risk factor ^1^	Number of studies included	N ^2^	Heterogeneity ^2^			Funnel plot asymmetry ( ***P*** -value) ^3^
			***I*** ^***2***^ (%) ^2^	***τ*** ^***2***^	***P*** -value	
FI (mU/L)	18	1756	99.5	3.762 ± 1.511	<0.001	0.519
FG (mmol/L)	24	3897	99.4	0.013 ± 0.005	<0.001	0.774
SBP (mm Hg)	23	3443	99.4	7.474 ± 2.894	<0.001	0.771
HDL (mmol/L)	30	4218	99.8	0.007 ± 0.002	<0.001	0.577
TAG (mmol/L)	29	3908	99.8	0.050 ± 0.015	<0.001	0.855
BMI (kg/m^2^)	29	4160	99.8	0.881 ± 0.256	<0.001	0.587

^1^ *FI*: Fasting insulin, *FG*: Fasting glucose, *SBP*: Systolic blood pressure, *HDL*: High density lipoprotein, *TAG*: Triacylglyceride, *BMI*: Body mass index.

^2^total number of participants, who concluded the interventions.

^2^ *I* ^*2*^ = heterogeneity (*τ* ^*2*^) expressed as a percentage of total variance (*τ* ^*2*^ + sample error).

^3^From Egger’s regression test.

### Intervention effect size estimates from mixed-effect models

Calorie intake restrictions, abnormalities such as IGT or IR, and resistance training exercises influenced FI responses to lifestyle interventions (*P* <0.10) and accounted for 60% of the heterogeneity [*τ*^2^ = 3.762 (Table 4) vs. 1.523 (Table 5)]. Lifestyle interventions reduced FI by 2.56 ± 0.58 mU/L (Table 5) in normal adults engaging in moderate intensity aerobic exercise. This decrease in FI was related to a mean 273 kcal/d restriction (Table 3). The negative parameter estimate of calorie restriction in Table 5 shows that an additional 100 kcal/d restriction would have reduced FI by an additional 0.68 ± 0.26 mU/L. On the other hand, incorporation of resistance training into the exercise intervention impeded the expected FI decline by 1.72 ± 0.81 mU/L. Hence, the expected FI change among adults engaging in resistance training was -0.84 mU/L (-2.56 + 1.72 mU/L). Regardless of the degree of calorie restriction and exercise intensity, adults with metabolic abnormalities tended (*P* = 0.082) to experience an extra FI reduction of 1.20 ± 0.69 mm Hg (Table 5).

**Table 5:** **Estimates of intervention effect size and total amount of residual heterogeneity (** ***τ*** ^**2**^ **) from final mixed-effect models**

Risk factor ^1^	Overall effect size		Effect size change for intervention attributes and other factors			Residual heterogeneity	
	Estimate ± SE	***P-*** value	Explanatory Variables ^2^	Estimate ± SE ^3^	***P*** -value	τ ^2^	***P*** -value
FI (mU/L)	-2.56 ± 0.58	<0.001	Presence of IGT, IR or MetS	-1.20 ± 0.69	0.082	1.523 ± 0.773	<0.001
			Calorie restriction (100 kcal/d)	-0.68 ± 0.26	0.009		
			Incorporation of resistance training	1.72 ± 0.81	0.034		
FG (mmol/L)	-0.18 ± 0.04	<0.001	Presence of IGT, IR or MetS	-0.08 ± 0.05	0.098	0.010 ± 0.004	<0.001
			After a follow-up period	0.13 ± 0.06	0.034		
SBP (mm Hg)	-2.77 ± 0.56	<0.001	Presence of IGT, IR or MetS	-3.23 ± 1.00	0.001	2.796 ± 1.419	<0.001
			Calorie restriction (100 kcal/d)	-0.64 ± 0.23	0.006		
			Length of intervention (months)	0.13 ± 0.03	<0.001		
HDL (mmol/L)	-0.015 ± 0.015	0.305	Age at baseline (years)	-0.006 ± 0.002	0.021	0.005 ± 0.002	<0.001
			Fraction of women participants	-0.086 ± 0.046	0.065		
TAG (mmol/L)	-0.258 ± 0.037	<0.001	Calorie restriction (100 kcal/d)	-0.061 ± 0.017	<0.001	0.029 ± 0.009	<0.001
			Baseline TAG (mmol/L)	-0.243 ± 0.076	0.001		
BMI (kg/m^2^)	-1.61 ± 0.13	<0.001	Calorie restriction (100 kcal/d)	-0.22 ± 0.08	0.003	0.353 ± 0.125	<0.001
			Fat intake restriction (% Energy)	-0.10 ± 0.03	0.003		
			Exercise session duration (10 min)	-0.24 ± 0.12	0.055		
			Baseline BMI (kg/m2)	-0.07 ± 0.04	0.095		

^1^ *FI*: Fasting insulin, *FG*: Fasting glucose, *SBP*: Systolic blood pressure, *HDL*: High density lipoprotein, *TAG*: Triacylglyceride, *BMI*: Body mass index.

^2^ *IGT* = Impaired glucose tolerance, *IR* = Insulin resistance and *MetS* = Metabolic syndrome.

^3^Standard error of the estimate.

Diet plus exercise interventions reduced FG by 0.18 ± 0.04 mmol/L (*P* <0.001) in normoglycemic healthy adults. The metabolic abnormality and follow-up period variables considerably explained the heterogeneity of the intervention effects on FG. Heterogeneity was reduced by 23% when these factors were included in the final model (*τ*^2^ = 0.013 vs. 0.010). Participants experienced less (by 0.13 ± 0.06 mmol/L) FG decline as they entered a follow-up period, during which rigorous intervention counselling was not provided. Regardless of undergoing a follow-up period or not, adults with metabolic abnormalities tended (*P* = 0.098) to experience a greater FG decline (by 0.08 ± 0.05 mmol/L) compared to normal adults (Table 5). Diet and exercise interventions reduced SBP by 2.77 ± 0.56 mm Hg (*P* <0.001, Table 5). However, study participants with abnormalities experienced an extra SBP decline of 3.23 ± 1.00 mm Hg (Table 5). Extending intervention by a month reduced the expected SBP decline by 0.13 ± 0.03 mm Hg. Calorie restriction increased by 100 kcal/d was associated with an additional SBP decline of -0.64 ± 0.23 mm Hg (*P* = 0.006). Presence or absence of abnormalities, intervention duration, and degree of calorie intake restriction explained collectively 63% [*τ*^2^ = 7.474 (Table 4) vs. 2.796 (Table 5)] of heterogeneity of the intervention effect on SBP.

The diet plus exercise interventions did not have a significant impact (*P* = 0.305) on HDL (Table 5). Nonetheless, age of participants at baseline and percentage of women in the study population explained 29% of heterogeneity in HDL changes in intervention participants compared to control participants. The lifestyle interventions reduced TAG by 0.258 ± 0.037 mmol/L (*P* <0.001). This drop in TAG was related to a mean net energy intake restriction of 273 kcal/d and a mean baseline TAG of 1.76 mmol/L (Table 3). An additional 100 kcal/d energy restriction would have further improved the decrease in TAG by 0.061 ± 0.017 mmol/L. A unit increase in TAG at baseline was associated with an additional TAG drop of 0.243 ± 0.076 mmol/L.

Adult participants experienced a 1.61 ± 0.13 kg/m^2^ (*P* <0.001) BMI decline when engaged in a diet plus exercise lifestyle intervention program. The extent of calorie and total fat intake restrictions, duration of exercise sessions, and baseline BMI considerably affected the degree of BMI change (*P* <0.060, Table 5). An extra 100 kcal/d energy intake restriction, a unit increase in total fat intake restriction and an additional 10 minutes of exercise would have further reduced BMI by 0.22 ± 0.08, 0.10 ± 0.03 and 0.24 ± 0.12 kg/m^2^, respectively. Obese adults appeared to respond more to the interventions than normal and overweight adults because a unit increase in BMI at baseline from the mean (30.1 kg/m^2^) was associated (*P* = 0.095) with an additional 0.07 ± 0.04 kg/m^2^ BMI decline. The final mixed-effect model including calorie and fat restrictions, exercise duration, and BMI at baseline explained 60% of the heterogeneity in lifestyle intervention effect on BMI [*τ*^2^ = 0.881 (Table 4) vs. 0.353 (Table 5)].

## Discussion

Although meta-analytic applications are increasingly used to summarize results from clinical trials, much uncertainty remains about which approach to use, particularly when significant between-study variability of results or heterogeneity is present [53]. Random-effect methods provide an attractive approach for summarizing heterogeneous results [53]. The lifestyle intervention effects of individual studies included in the present meta-analyses were very heterogeneous (Figures 2, 3 and 4). Therefore, the overall effect sizes and total amount of heterogeneity (*τ*^2^) was estimated using random-effect approaches. In addition to determining the overall effect size, random-effect approaches allow for exploring factors responsible for heterogeneity. These approaches involve mixed-effect models and are similar to meta-regression approaches. Thus, mixed-effect models were used to explore heterogeneity and estimate the impacts of important attributes of lifestyle interventions on risk factor changes. Such estimates are difficult to find in the literature but would improve diabetes risk prediction models. For example, Appuhamy and colleagues [54] developed a mathematical model for predicting diabetes incidence using BMI. Incorporating the effect size estimates of BMI (Table 5) should enable their model to predict diabetes incidence changes in response to lifestyle modifications. The present meta-analyses summarized effects of lifestyle modifications on other diabetes risk factors such as FI, FG, SBP, HDL and TAG. These factors are also used in mathematical models for predicting diabetes risk [5–7].

Fasting insulin in normoglycemic adults is an important predictor of diabetes risk independent of whether they have insulin resistance or not [55]. Thus, lowering FI could be an option in primary diabetes prevention [55]. The present meta-analyses showed that non-diabetic adults engaging in a diet plus exercise intervention program experienced a 17% reduction in FI from baseline. Although dietary guidelines in the studies advocate on average a 500 kcal/d energy restriction, the average net energy intake reduction among intervention participants was 273 kcal/d. A greater compliance with calorie restriction would have further decreased FI because FI was found to decrease linearly by 0.68 mU/L for each additional 100 kcal/d reduction. Hence an actual 500 kcal/d energy restriction would have reduced FI by 4.10 mU/L. Participants with impaired glucose tolerance or insulin resistance had their FI improved to a greater extent than normal participants. When resistance training was incorporated into moderate intensity aerobic exercises, the expected FI improvement for lifestyle interventions was significantly reduced by 1.72 mU/L. This agrees with Holten et al. [56] who demonstrated that strength training could increase FI levels in non-diabetic adults.

Fasting glucose is often used in diabetes risk prediction models [57], although plasma glucose based on the oral glucose tolerance test (OGTT) or the IGT test (IGTT) would better predict diabetes risk [58]. The greater cost and inconvenience associated with these tests generally impede their use in diabetes prediction models [59]. Furthermore, we chose FG over OGTT and IGTT-based glucose because FG was measured in many of the searched studies. Diet and exercise interventions were associated with a decline in FG of 0.18 mmol/L, representing a 3% drop from baseline (*P* <0.001). Elevating FG from normoglycemic levels to impaired fasting glucose (IFG) levels doubles the risk of developing diabetes [60, 61]. The mean baseline FG of 5.55 mmol/L indicates that many of the study participants were at a high risk of developing diabetes especially considering the fact that the current cutoff for IFG (5.60 mmol/L) needs to be reduced for some Western populations [14]. Therefore, this 3% FG reduction should delay diabetes development in Western adults. Our results showed that continuous dietary counselling and physical activity improvement were necessary for a persistent FG decline. Moreover, the FG levels of adults with abnormalities such as IGT or MetS improved more than those of normal adults.

Hypertension is recognized as an independent predictor of diabetes incidence in various populations. Systolic blood pressure above 120 mm Hg is associated with twice the diabetes risk of SBP below 100 mm Hg [62]. The present meta-analyses showed that diet plus exercise intervention counseling reduced (*P* <0.001) SBP by 2.77 mm Hg. This decline in SBP could be associated with a considerable reduction in diabetes risk as Dotevall et al. [63] reported a 0.10 diabetes hazard ratio increase for every unit increase in SBP above 130 mm Hg. Normoglycemic adults with abnormalities such as IGT or MetS experienced greater declines in SBP, suggesting more lifestyle intervention benefits for them than for ordinary adults. An improved compliance with calorie restriction guidelines, particularly as the intervention program progressed, could have resulted in a greater SBP decline.

Dyslipidaemia, characterized by elevated TAG and reduced HDL, is usually associated with increased risk of developing diabetes mellitus [64]. Diet plus exercise interventions reduced TAG by 0.258 mmol/L (*P* <0.001). This could be associated with a considerably reduced risk of developing diabetes as Tirosh et al. [65] reported a 13% reduction in diabetes risk for each 0.20 mmol/L decline in TAG in non-diabetic men. The declines in TAG were greater as baseline TAG levels increased, suggesting more lifestyle intervention benefits for dyslipidaemic adults than normolipidaemic adults. Greater calorie restrictions further enhanced the TAG declines, in agreement with the findings of Fontana et al. [66]. When summarized over 30 studies, diet plus exercise interventions did not have a significant impact on HDL. Fourteen studies were associated with reduced HDL (the negative mean differences in Figure 4A) while the rest showed zero or positive responses. Differences in baseline age and percentage of women in the study population explained 29% of the heterogeneity in HDL responses to lifestyle interventions.

Body mass index is a leading diabetes risk predictor as evidenced by the high correlation between obesity and diabetes prevalence. Curioni and Lourenco [67] and Schaar et al. [68] have summarized diet plus exercise intervention effects on weight loss in adults regardless of diabetes status. These analyses together examined 22 studies and found significant weight loss in response to intervention. We found a consistent reduction in BMI (*P* <0.001) of 1.61 kg/m^2^ (5.3% from baseline BMI) for diet plus exercise interventions. Such a BMI reduction could be associated with a considerable reduction in diabetes risk as Chiu et al. [69] estimated a 30% diabetes incidence drop in response to a BMI reduction from 30.0 to 28.0 kg/m^2^. Moreover, the mathematical model developed by Appuhamy et al. [53] predicts that a 1.61 kg/m^2^ in BMI decline can lead to a 20% diabetes incidence reduction in non-diabetic middle-aged adults. The heterogeneity in lifestyle intervention effect on BMI was significant (Figure 2B and Table 4). However, degrees of calorie and total fat intake restrictions, duration of exercise and BMI at baseline explained 60% of the heterogeneity. An additional 100 kcal/d calorie intake reduction, an extra unit reduction in total fat intake, and extending the exercise session length by 10 minutes could collectively result in an additional 0.56 kg/m^2^ decline in BMI. BMI decline significantly improved as baseline BMI increased, suggesting greater lifestyle intervention benefit for obese adults than for adults of normal weight.

Considerable unexplained heterogeneity still remained (residual heterogeneity estimates in Table 5), indicating that factors other than those taken into consideration in the present analyses could be responsible for between-study variability of lifestyle intervention effects. In addition to carbohydrate, fat and protein as explanatory variables, the present meta-analyses also accounted for saturated fat and cholesterol intake restrictions, and fiber intake improvements. However, they did not significantly (*P* >0.10) explain heterogeneity of lifestyle intervention effects. Data on food sources of fatty acids could have explained heterogeneity to some extent [70, 71] but availability of such data was extremely limited in the studies considered. Representations of exercise intensity (e.g., percent maximum heart rate and percent maximum oxygen consumption) may have explained some of the heterogeneity. Furthermore, representation of degree of compliance with diet and exercise guidelines [72] and ethnic composition of participants [73] could have further explained heterogeneity. As the selected articles [20–49] did not consistently report sufficient information to create such variables, we were unable to explore their contribution to heterogeneity.

In summary, lifestyle interventions targeting calorie and total fat intake restrictions and increase in moderate intensity aerobic exercises were associated with significant improvements in diabetes risk factors among normoglycemic adults. Differences in some intervention attributes such as energy and fat intake restrictions, exercise type and session duration, length of intervention, and baseline characteristics of study participants accounted for 23-63% of the heterogeneity. Having explained such proportions of heterogeneity, lifestyle interventions were associated with significant declines in FI, FG, SBP, TAG, and BMI of 2.56 mU/L, 0.18 mmol/L, 2.77 mm Hg, 0.258 mmol/L, and 1.61 kg/m^2^ respectively in healthy normoglycemic adults. However, normoglycemic adults having abnormalities such as IGT, insulin resistance, metabolic syndrome or hyperlipidemia appeared to benefit more from diet plus exercise intervention programs than healthy normoglycemic adults.
